# Microscale and Nanoscale Biosensors

**DOI:** 10.3390/bios8030066

**Published:** 2018-07-06

**Authors:** Beatriz Jurado-Sánchez

**Affiliations:** 1 Department of Analytical Chemistry, Physical Chemistry and Chemical Engineering, University of Alcalá, E-28871 Alcala de Henares, Madrid, Spain; beatriz.jurado@uah.es; Tel.: +34-918-854-941; 2 Chemical Research Institute “Andrés M. del Río”, University of Alcala, E-28871 Alcala de Henares, Madrid, Spain

**Keywords:** biosensor, filter paper, microfluidic, cell monitoring, S-layer protein, micromotors

## Abstract

The emerge of nanotechnology along with the success of the microelectronics industry has motivated the miniaturization of biosensors into the nano/microscale. This Special Issue highlights recent advances in microscale and nanoscale biosensors, including self-propelled micromotors: their materials, fabrication, and applications. A total of seven papers (five research and two review papers) are included. Different but related topics are covered, from biosensor design (paper strips and digital microfluidic chips) to integrated configurations that monitor metabolites in cellular environments. The reviews are devoted to protein-based biosensors and moving biosensors based on self-propelled micromotors.

This Special Issue aims to highlight the most recent and promising technologies in micro and nanoscale biosensors, including self-propelled micromotors: their materials, fabrication, and applications. Current progress in nanoscience and nanotechnology has paved the way for the design of many new multi-functional materials for (bio)sensing purposes. Modern biosensors, based on such microscale and nanoscale materials, offer new opportunities in analytical chemistry, healthcare, and many other fields. Indeed, the reduction in size to nanoscale dimensions may result in cheaper, portable, and easier-to-use analytical tools, allowing for real-time analytical measurements, detection in microfluidic systems, and in vivo monitoring applications. Of great interest, self-propelled micromotors represent a new paradigm in the field as novel nanoscale biosensors for the development of ‘on-the-move’ sensing schemes.

This Special Issue compromise five research papers, one feature, and one review article. Two research papers explore different configurations (paper strips and digital microfluidic chips) for biosensing purposes. In the first article, Matějovský and Pitschmann [1] illustrate a simple “paper strip” biosensor based on the Ellman´s reagent for the visual detection of chemical warfare agents (Sarin and VX types) in air, water, aqueous homogenates, food, soil, and on the surface of objects. Zulkepli et al. [2] described a more sophisticated biosensing design based on digital microfluidic biochips. Such miniaturized systems rely on a dielectric layer to allow microliter droplets movement based on the electrowetting-on-dielectric (EWOD) technique. The so-called OpenDrop device was tested by utilizing a single-plate system to transport microliter droplets for a bioassay operation, holding considerable promise for basic scientific research and medical diagnostics devices. 

A second important core of the Special Issue deals with the integration of biosensors in cell environments to monitor metabolites associated with cell events. Kieninger et al. [3] employed microfabricated sensor chips embedded in standard cell culture flasks equipped with amperometric oxygen sensors. The utility was demonstrated to monitor cellular respiration in cultured brain tumor (T-98G) and breast cancer (T-47D) cells. Cellular acidification was accessed with potentiometric pH sensors using electrodeposited iridium oxide films. Nasr et al. [4] developed a nanostructured electrode by the functionalization of borosilicate glass capillaries as electrochemical biosensors to detect glutamate release from cerebral organoids generated from human embryonic stem cells. Such an important metabolite can be an indicator of neuronal maduration. Chistyakov et al. [5] used Escherichia coli MG1655 (pRecA-lux) to evaluate the ability of the fermenters of eight probiotic strains to reduce the SOS response stimulated by ciprofloxacin in bacteria, and the mutagenesis mediated by it. 

The Special Issue also compromises a feature article devoted to S-layer protein biosensors [6], which highlights the application of bacterial surface (S-) layer proteins as versatile components for the fabrication of biosensors. To conclude, a review article [7] gives a comprehensive overview of self-propelled micro and nanomotors as the next generation of “moving” biosensors. Indeed, micro/nanomotors hold considerable potential for developing novel biosensing protocols involving ‘on-the-move’ recognition and sensing events, overcoming major challenges in biosensor miniaturization, such as an adequate trade-off between sensor dimensions–signal transduction efficiency and reaction transport kinetics. 
